# Variation in the rates of emergency surgery amongst emergency admissions to hospital for common acute conditions

**DOI:** 10.1093/bjsopen/zrab094

**Published:** 2021-11-17

**Authors:** Andrew Hutchings, Silvia Moler Zapata, Stephen O’Neill, Neil Smart, David Cromwell, Robert Hinchliffe, Richard Grieve

**Affiliations:** 1 Department of Health Services Research and Policy, London School of Hygiene & Tropical Medicine, London, UK; 2 College of Medicine and Health, University of Exeter, Exeter, UK; 3 Clinical Effectiveness Unit, Royal College of Surgeons, London, UK; 4 Bristol Surgical Trials Centre, University of Bristol, Bristol, UK

## Abstract

**Background:**

This paper assesses variation in rates of emergency surgery (ES) amongst emergency admissions to hospital in patients with acute appendicitis, cholelithiasis, diverticular disease, abdominal wall hernia, and intestinal obstruction.

**Methods:**

Records of emergency admissions between 1 April 2010 and 31 December 2019 for the five conditions were extracted from Hospital Episode Statistics for 136 acute National Health Service (NHS) trusts in England. Patients who had ES were identified using Office of Population Censuses and Surveys (OPCS) procedure codes, selected by consensus of a clinical panel. The differences in ES rates according to patient characteristics, and unexplained variations across NHS trusts were estimated by multilevel logistic regression, adjusting for year of emergency admission, age, sex, ethnicity, diagnostic subcategories, index of multiple deprivation, number of co-morbidities, and frailty.

**Results:**

The cohort sizes ranged from 107 325 (hernia) to 268 253 (appendicitis) patients, and the proportion of patients who received ES from 11.0 per cent (diverticular disease) to 92.3 per cent (appendicitis). Older patients were generally less likely to receive ES, with adjusted odds ratios (ORs) of ES for those aged 75–79 *versus* those aged 45–49 years: 0.34 (appendicitis), 0.49 (cholelithiasis), 0.87 (hernia), and 0.91 (intestinal obstruction). Patients with diverticular disease aged 75–79 were more likely to receive ES than those aged 45–49 (OR 1.40). Variation in ES rates across NHS trusts remained after case mix adjustment and was greatest for cholelithiasis (trust median 18 per cent, 10th to 90th centile 7–35 per cent).

**Conclusion:**

For patients presenting as emergency hospital admissions with common acute conditions, variation in ES rates between NHS trusts remained after adjustment for demographic and clinical characteristics. Age was strongly associated with the likelihood of ES receipt for some procedures.

## Teaser

This study reports variation in rates of emergency surgery (ES) across National Health Service trusts amongst patients presenting as emergency admissions to hospital with acute appendicitis, cholelithiasis, diverticular disease, abdominal wall hernia, or intestinal obstruction, after adjustment for differences in patient-level characteristics. Older patients were less likely to have ES, after allowing for differences in other patient characteristics, including number of Charlson co-morbidities, level of frailty, and diagnostic subcategory. The decline in the rate of ES with increasing age was greatest for patients presenting with acute appendicitis and cholelithiasis.

## Introduction 

Emergency surgery (ES) poses a considerable global burden to publicly funded health systems^1^, and is responsible for approximately 750 000 admissions per year in England alone^2^, with surgical procedures accounting for approximately 10 per cent of the annual National Health Service (NHS) budget^3^. In the USA, there are around three million hospital admissions presenting for ES, at an estimated cost of $28 billion, projected to rise to about $41 billion by 2060^4^. For common acute conditions (for example, acute diverticular disease) that present as emergency admissions, an area of ongoing concern is which patients should receive ES *versus* non-emergency surgery (NES) strategies that include medical management, non-surgical procedures (for example, drainage of abscess), and surgery deferred to the elective (planned) setting.

For patients with acute conditions, ES rates have declined over the last two decades^5^, which may reflect changes in the characteristics of those presenting as emergency admissions and improved diagnostics. In addition, protocols for NES strategies have been implemented as part of RCTs, and for some acute conditions, these have resulted in outcomes similar to those of ES^6^^,^^7^. During the coronavirus disease 2019 (COVID-19) pandemic, international guidelines have recognized further reductions in ES rates for acute conditions^1^. However, delaying or avoiding surgical strategies may have unintended consequences^8^^,^^9^. Patients with acute conditions, such as acute cholelithiasis or inguinal hernia, who do not have ES can develop severe complications such as acute pancreatitis or strangulated bowel, or have recurrent symptoms leading to delayed surgery and further pressure on surgical waiting lists^8^^,^^10^. Despite initiatives to standardize the clinical management of these acute conditions^11–13^, clinical uncertainty about the benefits and harms of ES, as well as the difference in availability of surgical facilities and specialists, may lead to wide variations in ES for patients with common acute conditions. However, previous evidence of variation in ES across NHS trusts in England has been limited to a single condition or a short time period^14^, or has not recognized the role of patient factors such as frailty or the number of co-morbidities^11–13^.

The aim of this study was to investigate the association between patient factors such as age with ES, and the unexplained variation in ES in adults across NHS hospital trusts in England from 2010 to 2019, for emergency hospital admissions with common acute conditions.

## Methods

This National Institute for Health Research (NIHR)-funded study Emergency Surgery or Not (ESORT) uses national Hospital Episodes Statistics (HES) data for England to define patient cohorts admitted as emergencies to NHS acute hospital trusts for five common acute conditions: appendicitis, cholelithiasis, intestinal obstruction (small or large bowel), (symptomatic) diverticular disease, and abdominal wall hernia^15^. These acute conditions were defined according to the International Classification of Diseases, tenth revision (ICD-10) diagnosis codes corresponding to each condition.

The research was approved by the London School of Hygiene and Tropical Medicine ethics committee (Ethics Reference no.: 21687). The study involved secondary analyses of existing pseudo-anonymized data and did not require UK National Ethics Committee approval. The study drew from the findings of two workshops with patients and the public, held in July 2020, that reported it was of potential benefit to patients and the public to examine why access to ES might vary according to different patient groups^16^.

### Study population

An admission can comprise several finished consultant episodes, and patients aged 18 or over were eligible for inclusion in the study cohort if a finished consultant episode met the following criteria: occurred between 1 April 2010 and 31 December 2019; included a main diagnosis with an ICD-10 diagnosis code (*Tables S1 and S2*) that was deemed relevant according to the consensus of a clinical panel; was within an emergency admission through the emergency department or was from a primary care referral; was under a consultant general surgeon, subspecialty general surgeon, or surgeon working in the general surgery specialty; and was the first or second episode within the admission. For the intestinal obstruction cohort, a relevant diagnosis could appear in the second diagnosis field if the main diagnosis was colorectal cancer. An admission was excluded if there had been a prior emergency admission with a relevant diagnosis in the previous 12 months, or further diagnostic exclusion criteria were met according to the consensus of a clinical panel (see *Table S2*).

### Definition of emergency surgery

The final list of procedures, as defined according to Office of Population Censuses and Surveys (OPCS) codes, and the maximum number of days within which the surgery had to occur to constitute ES were defined by consensus of the study’s clinical panel (for full details, see *Table S3*, and weblink^17^). In brief, the qualifying surgical procedure had to be within 3 days (hernia), 7 days (appendicitis, cholelithiasis, intestinal obstruction), or any time within the emergency admission (diverticular disease).

### Patient characteristics and definition of NHS trusts

The following patient characteristics were available from HES data at admission and considered to potentially influence the treatment decision: age (years), sex, ethnicity, Index of Multiple Deprivation (IMD), diagnostic subcategories, number of co-morbidities, and frailty. The Charlson co-morbidity index^18^ and secondary care administrative records frailty (SCARF) index frailty index^19^ were derived for all patients. The frailty index is based on a cumulative deficit model of frailty and uses the International Statistical Classification of Diseases, Injuries, and Causes of Death, 10th revision codes to define a set of 32 deficits that cover functional impairment, geriatric syndromes, problems with nutrition, cognition, and mood, and medical co-morbidities^19^. Those patients with missing ethnicity data were designated to a missing data category. The proportion of qualifying admissions who had ES were derived for 136 general acute NHS trusts in existence on 31 March 2016. Organizational changes during the study period, such as trust mergers, were addressed by mapping 175 hospitals to their 2016 NHS trust status. The total volume of emergency admissions that met the inclusion criteria for each trust was calculated over the time period.

### Statistical analysis

Summary statistics were used to describe patients’ demographic and clinical characteristics. Age was categorized into 5-year age bands. The proportions of eligible emergency admissions were calculated for each cohort overall, and according to prespecified subgroups of interest. For each condition, multilevel logistic models were developed which included year of emergency admission, age, sex, ethnicity, diagnostic subcategories, IMD (quintiles), number of Charlson co-morbidities, and SCARF frailty index as independent variables, and whether the patient received ES or not as the dependent variable. The unit of analysis was the emergency admission. The multilevel model included random intercepts for each NHS trust to allow for clustering and to report the level of unexplained trust-level variation in ES, after allowing for patient factors and time period. The model was used to predict the case mix adjusted odds ratios (95per cent confidence interval) of ES associated with patient factors, and the levels of unexplained variation attributable to NHS trusts. Funnel plots were used to display the variation in case mix adjusted proportions receiving ES *versus* the volume of emergency admissions within NHS trusts.

## Results

### Patient characteristics

The number of patients who were eligible as emergency admissions were: 107 325 (hernia), 137 744 (intestinal obstruction), 139 090 (diverticular disease), 241 626 (cholelithiasis), and 268 253 (appendicitis) (*Table S4*). *Table 1* presents the patient characteristics for each cohort of emergency admissions that met the study’s inclusion criteria. The numbers (proportions) of patients in each diagnostic subcategory are listed in *Table S5*.

**Table 1:** Patient characteristics of the five cohorts

	Appendicitis	Cholelithiasis	Diverticular disease	Hernia	Intestinal obstruction
	(*n* = 268 253)	(*n* = 241 626)	(*n* = 139 090)	(*n* = 107 325)	(*n* = 137 744)
**Age category: *n* (%)**					
Under 25	63 405 (23.6)	12 137 (5.0)	310 (0.2)	2282 (2.1)	2251 (1.6)
25–29	37 585 (14.0)	15 339 (6.4)	1077 (0.8)	3159 (2.9)	2352 (1.7)
30–34	31 391 (11.7)	16 480 (6.8)	2471 (1.8)	4021 (3.8)	2807 (2.0)
35–39	25 494 (9.5)	16 121 (6.7)	4659 (3.4)	4760 (4.4)	3520 (2.6)
40–44	21 668 (8.1)	17 783 (7.4)	7595 (5.5)	6137 (5.7)	4770 (3.5)
45–49	19 799 (7.4)	20 627 (8.5)	11 482 (8.3)	7832 (7.3)	6850 (5.0)
50–54	17 431 (6.5)	21 133 (8.8)	14 021 (10.1)	8295 (7.7)	8578 (6.2)
55–59	13 844 (5.2)	19 783 (8.2)	14 077 (10.1)	8014 (7.5)	9724 (7.1)
60–64	11 158 (4.2)	18 907 (7.8)	13 681 (9.8)	8406 (7.8)	11 612 (8.4)
65–69	9464 (3.5)	19 799 (8.2)	14 339 (10.3)	9241 (8.6)	14 462 (10.5)
70–74	6992 (2.6)	18 969 (7.9)	14 677 (10.6)	10 414 (9.7)	16 425 (11.9)
75–79	4729 (1.8)	16 863 (7.0)	14 106 (10.1)	10 859 (10.1)	17 330 (12.6)
80–84	3019 (1.1)	14 179 (5.9)	12 893 (9.3)	10 881 (10.1)	16 686 (12.1)
85–89	1606 (0.6)	9061 (3.8)	9149 (6.6)	8276 (7.7)	12 697 (9.2)
90 and over	668 (0.3)	4445 (1.8)	4553 (3.3)	4748 (4.4)	7680 (5.6)
**Sex: *n* (%)**					
Female	123 520 (46.0)	163 219 (67.6)	81 994 (59.0)	37 776 (35.2)	72 237 (52.4)
Male	144 720 (54.0)	78 398 (32.4)	57 093 (41.0)	69 545 (64.8)	65 504 (47.6)
Missing	13	9	3	4	3
**Ethnicity: *n* (%)**					
Black/black mixed	6401 (2.7)	4761 (2.1)	2132 (1.6)	2647 (2.7)	3433 (2.6)
Asian/Asian mixed	12 721 (5.3)	11 359 (5.0)	2421 (1.8)	3621 (3.6)	4462 (3.4)
White	211 433 (88.0)	207 696 (90.7)	126 246 (95.2)	91 651 (91.7)	122 152 (92.3)
Chinese and other	9764 (4.1)	5105 (2.2)	1876 (1.4)	1989 (2.0)	2361 (1.8)
Missing	27 934	12 705	6415	7417	5336
**Deprivation quintile**					
Most deprived	53 835 (20.4)	56 610 (23.7)	25 024 (18.1)	23 033 (21.7)	24 167 (17.7)
2	54 385 (20.6)	50 779 (21.2)	27 325 (19.8)	22 094 (20.8)	26 253 (19.3)
3	53 351 (20.2)	48 313 (20.2)	29 119 (21.1)	21 908 (20.6)	28 914 (21.2)
4	51 739 (19.6)	44 492 (18.6)	29 270 (21.2)	20 646 (19.4)	28 796 (21.1)
Least deprived	50 564 (19.2)	39 067 (16.3)	27 180 (19.7)	18 614 (17.5)	28 092 (20.6)
Missing	4379	2365	1172	1030	1522
**Co-morbidity: *n* (%)**					
None	222 935 (83.1)	157 866 (65.3)	83 367 (59.9)	66 156 (61.6)	72 308 (52.5)
1	39 727 (14.8)	62 343 (25.8)	39 661 (28.5)	29 847 (27.8)	43 582 (31.6)
2	4753 (1.8)	17 108 (7.1)	12 697 (9.1)	9013 (8.4)	17 129 (12.4)
3 or more	838 (0.3)	4309 (1.8)	3365 (2.4)	2309 (2.2)	4725 (3.4)
**Frailty index: *n* (%)**					
Fit	221 900 (82.7)	157 866 (65.3)	72 225 (51.9)	57 435 (53.5)	62 989 (45.7)
Mild frailty	38 612 (14.4)	62 343 (25.8)	44 551 (32.0)	32 973 (30.7)	45 428 (33.0)
Moderate frailty	6200 (2.3)	17 108 (7.1)	16 163 (11.6)	12 416 (11.6)	20 497 (14.9)
Severe frailty	1541 (0.6)	4309 (1.8)	6151 (4.4)	4501 (4.2)	8830 (6.4)

### Receipt of ES

The proportion of patients who had ES was highest for acute appendicitis (92.3per cent), and 57.9 per cent for hernia, 29.9 per cent for intestinal obstruction, 21.5 per cent for cholelithiasis, and 11.0 per cent for diverticular disease (*Table S4*). The most common ES procedures are listed in *Table S6*. The proportion of patients who received ES was generally lower for older age groups (*Table 2*). Women were more likely than men to receive ES for cholelithiasis, hernia, and intestinal obstruction. For all five conditions, patients with co-morbidities were less likely to receive ES than those without. The proportion of patients who had ES increased with frailty for patients with diverticular disease and intestinal obstruction but decreased for those with appendicitis and hernia.

**Table 2:** Percentage of emergency admissions receiving ES according to patient characteristics

	Appendicitis	Cholelithiasis	Diverticular disease	Hernia	Intestinal obstruction
	(*n* = 268 253)	(*n* = 241 626)	(*n* = 139 090)	(*n* = 107 325)	(*n* = 137 744)
**Age category**					
Under 25	99.0	26.4	14.5	50.2	32.5
25–29	94.5	27.4	13.0	51.9	27.3
30–34	94.0	26.3	11.5	53.7	27.8
35–39	93.8	25.6	10.9	53.6	29.4
40–44	93.1	25.0	10.9	55.6	30.9
45–49	92.0	24.8	9.9	57.2	31.4
50–54	91.3	24.1	9.6	58.4	30.2
55–59	89.9	23.0	9.7	58.5	31.1
60–64	88.4	20.7	11.4	59.4	31.2
65–69	86.7	19.9	13.1	61.4	31.7
70–74	85.3	18.2	13.5	60.6	31.8
75–79	80.5	15.8	12.9	60.0	30.9
80–84	75.5	12.4	11.3	59.2	30.5
85–89	67.3	9.8	8.1	57.8	26.3
90 and over	50.8	6.8	4.2	53.0	19.6
**Sex**					
Female	91.7	22.6	10.3	65.5	32.6
Male	92.8	19.3	12.0	53.8	26.8
**Co-morbidity index**					
None	93.1	23.3	11.4	59.1	31.6
1	89.9	19.7	10.9	58.0	29.9
2	78.1	14.9	9.2	52.6	24.8
3 or more	65.0	10.1	8.1	45.2	21.0
**Frailty index**					
Fit	93.2	22.8	8.3	57.2	28.2
Mild frailty	89.4	20.5	12.2	59.1	30.6
Moderate frailty	81.3	17.3	16.2	58.5	32.2
Severe frailty	73.0	15.6	19.4	56.5	32.5

### Patient factors associated with ES receipt

**Fig. 1:**
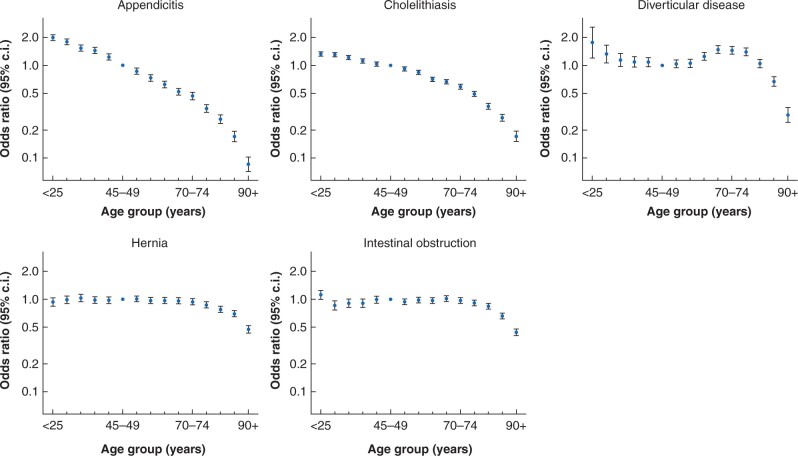
Association between age group and ES receipt, with adjusted odds ratios (95% confidence intervals) in comparison with 45- to 49-year olds

*Table 3* presents the association of each patient factor with ES rate, after adjustment for other patient-level variables, hospital trust, and time period. For all conditions, except diverticular disease, after adjustment, ES rates declined for older age groups, with adjusted odds ratios for patients aged 85–89 years *versus* those aged 45–49 years ranging from 0.2 (appendicitis) to 0.68 (hernia). The decline in ES rates with increasing age was sharpest for appendicitis and cholelithiasis. For patients with diverticular disease, ES rates were higher for patients aged 60–80 years (*Fig. 1*). After adjusting for frailty and other factors, patients with any of the five conditions were less likely to have ES if they had co-morbidities. The association of frailty with ES rate differed by condition. There was a consistent decline in the rate of ES as the number of Charlson co-morbidities increased, but the association between frailty and ES rate was less consistent. The relationship was strongest for patients with acute appendicitis or hernia; those patients with more severe frailty were less likely to receive ES. Patients at any level of frailty who presented with cholelithiasis, diverticular disease, or intestinal obstruction were more likely to receive ES (*Table 3*). Investigation of interactions between co-morbidity and frailty showed that in diverticular disease and intestinal obstruction, the effect of frailty was strongest in patients with no Charlson co-morbidities (*Table S7*).

**Table 3:** Emergency admissions receiving ES according to patient characteristics, with adjusted odds ratios

	Appendicitis	Cholelithiasis	Diverticular disease	Hernia	Intestinal obstruction
	(*n* = 268 253)	(*n* = 241 626)	(*n* = 139 090)	(*n* = 107 325)	(*n* = 137 744)
**Age category**					
Under 25	2.00 (1.87, 2.13)	1.33 (1.25, 1.40)	1.76 (1.20, 2.60)	0.93 (0.84, 1.04)	1.11 (1.00, 1.25)
25–29	1.79 (1.67, 1.92)	1.30 (1.24, 1.37)	1.33 (1.06, 1.66)	0.99 (0.90, 1.09)	0.86 (0.77, 0.96)
30–34	1.54 (1.43, 1.66)	1.22 (1.15, 1.28)	1.14 (0.97, 1.34)	1.03 (0.94, 1.12)	0.91 (0.82, 1.01)
35–39	1.44 (1.34, 1.56)	1.12 (1.06, 1.18)	1.09 (0.96, 1.24)	0.98 (0.90, 1.06)	0.91 (0.82, 1.00)
40–44	1.23 (1.14, 1.32)	1.03 (0.98, 1.09)	1.08 (0.97, 1.21)	0.97 (0.90, 1.05)	0.99 (0.91, 1.08)
45–49	Reference	Reference	Reference	Reference	Reference
50–54	0.87 (0.80, 0.94)	0.92 (0.87, 0.96)	1.04 (0.94, 1.14)	1.01 (0.94, 1.08)	0.94 (0.87, 1.01)
55–59	0.73 (0.68, 0.79)	0.84 (0.80, 0.89)	1.05 (0.96, 1.16)	0.97 (0.90, 1.04)	0.98 (0.91, 1.06)
60–64	0.62 (0.57, 0.68)	0.71 (0.67, 0.74)	1.25 (1.13, 1.37)	0.97 (0.90, 1.04)	0.97 (0.91, 1.04)
65–69	0.52 (0.48, 0.57)	0.66 (0.63, 0.70)	1.48 (1.34, 1.62)	0.96 (0.89, 1.03)	1.01 (0.95, 1.08)
70–74	0.47 (0.43, 0.51)	0.59 (0.56, 0.62)	1.45 (1.32, 1.60)	0.94 (0.87, 1.01)	0.96 (0.90, 1.03)
75–79	0.34 (0.31, 0.38)	0.49 (0.46, 0.52)	1.40 (1.27, 1.54)	0.87 (0.81, 0.93)	0.91 (0.85, 0.97)
80–84	0.26 (0.24, 0.29)	0.36 (0.34, 0.39)	1.05 (0.94, 1.16)	0.78 (0.72, 0.84)	0.84 (0.79, 0.90)
85–89	0.17 (0.15, 0.19)	0.27 (0.25, 0.29)	0.67 (0.59, 0.75)	0.70 (0.64, 0.75)	0.66 (0.61, 0.71)
90 and over	0.09 (0.07, 0.10)	0.17 (0.15, 0.19)	0.29 (0.24, 0.35)	0.47 (0.43, 0.52)	0.44 (0.40, 0.48)
**Sex**					
Female	0.95 (0.93–0.98)	1.18 (1.15–1.21)	0.97 (0.93, 1.02)	0.98 (0.95, 1.02)	1.27 (1.24, 1.30)
Male	Reference	Reference	Reference	Reference	Reference
**Co-morbidity**					
None	Reference	Reference	Reference	Reference	Reference
1	0.86 (0.82, 0.90)	0.87 (0.84, 0.89)	0.71 (0.68, 0.75)	0.88 (0.85, 0.91)	0.77 (0.75, 0.80)
2	0.54 (0.50, 0.59)	0.67 (0.64, 0.71)	0.44 (0.41, 0.48)	0.71 (0.66, 0.75)	0.56 (0.54, 0.59)
3 or more	0.37 (0.31, 0.43)	0.44 (0.39, 0.49)	0.33 (0.28, 0.38)	0.57 (0.51, 0.63)	0.42 (0.39, 0.46)
**Frailty index**					
Fit	Reference	Reference	Reference	Reference	Reference
Mild	0.99 (0.94, 1.03)	1.11 (1.08, 1.15)	2.00 (1.90, 2.11)	1.01 (0.97, 1.05)	1.42 (1.38, 1.47)
Moderate	0.89 (0.81, 0.97)	1.22 (1.16, 1.28)	3.29 (3.06, 3.54)	0.93 (0.88, 0.99)	1.84 (1.77, 1.92)
Severe	0.80 (0.70, 0.92)	1.34 (1.23, 1.45)	3.84 (3.48, 4.24)	0.72 (0.66, 0.78)	1.98 (1.87, 2.10)

### Variation in ES rates across NHS trusts

**Fig. 2:**
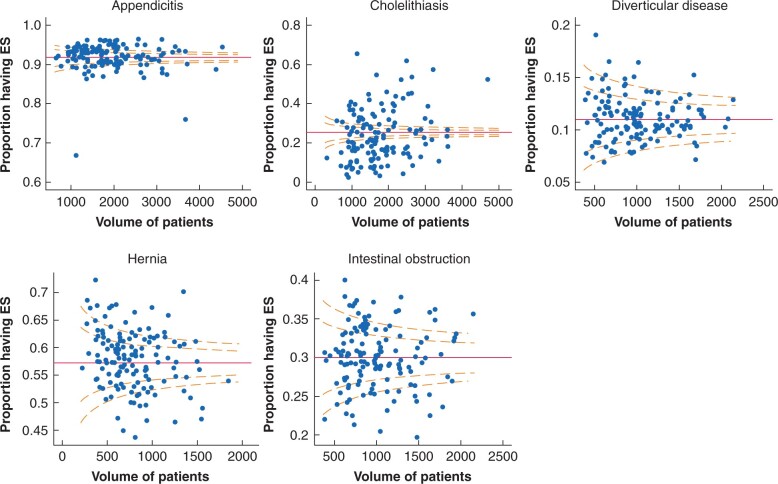
Funnel plots of variation in rates of emergency surgery in 136 acute NHS trusts in England from April 2010 to December 2019

Before case mix adjustment, the overall variation in ES rates across NHS trusts was greatest for cholelithiasis (median of 18.9per cent, 10th to 90th centile 7.1–35.4per cent) and hernia (59.0per cent, 49.9–69.6per cent), followed by intestinal obstruction (29.9per cent, 24.3–35.6per cent), appendicitis (93.0per cent, 88.5–96.1per cent), and diverticular disease (10.9per cent, 7.9–15.0per cent). *Fig. 2* shows that variation in ES rates across NHS trusts remained after case mix adjustment. Rates of ES between trusts were positively correlated for all conditions and highest between appendicitis and cholelithiasis (*r* = 0.33). The level of unexplained variation did not appear related to the volume of emergency admissions for the respective condition within each trust. The estimated proportion of unexplained variation at the level of the NHS trust, rather than of the patient, was highest for cholelithiasis, with intraclass correlation (95per cent confidence interval) of 0.169 (0.137 to 0.205), followed by appendicitis (0.053, 0.042 to 0.067), hernia (0.027, 0.021 to 0.035), diverticular disease (0.022, 0.016 to 0.029), and intestinal obstruction (0.014, 0.010 to 0.018).

## Discussion

This report describes variation in rates of ES across NHS trusts for patients presenting as emergency admissions to hospital with acute appendicitis, cholelithiasis, diverticular disease, abdominal wall hernia, or intestinal obstruction. This variation remained after adjustment for differences in patient-level characteristics and the time period of the emergency admission, and was greatest for patients admitted as an emergency with a diagnosis of acute cholelithiasis. The study also reported wide differences in ES rates according to age group. Older patients were less likely to receive ES, after allowing for differences in other patient characteristics, including number of Charlson co-morbidities, level of frailty, and diagnostic subcategory. The decline in the rate of ES with increasing age was highest for patients admitted as an emergency with appendicitis and cholelithiasis.

The study found differences amongst the five conditions in the levels of unexplained variation across NHS trusts in ES rates. The highest level of unexplained variation in ES rates was for patients presenting with acute cholelithiasis, which suggests that some trusts did not follow the National Institute for Health and Care Excellence (NICE) guidelines recommending laparoscopic cholecystectomy within 7 days of diagnosis^20^. These guidelines are informed by evidence from a meta-analysis that reported improved outcomes with ES *versus* delayed cholecystectomy for patients with biliary colic, acute cholecystitis, and gallstone pancreatitis^21^. Despite these recommendations, related research has also reported high levels of unexplained variation across NHS trusts in ES rates over a 2-month period^14^. The present study adds to these previous findings in reporting these levels of unexplained variation across a large number (136) of NHS trusts over a 10-year time period.

The unexplained variation in ES rates for patients with acute cholelithiasis was obtained after adjusting for the annual volume of ES procedures performed in each trust and may reflect differences in the level of surgical expertise and resource availability across NHS trusts. Previous research on trust-level variation for patients with benign gallbladder diseases reported higher rate of ES in those centres with a specialist hepatobiliary centre available, which may reflect better availability of operating theatre space, clearer understanding of the evidence comparing emergency *versus* delayed cholecystectomy, or the enthusiasm to deliver an emergency cholecystectomy service^14^. This previous study found that other trust-level factors, such as availability of ES operating lists specific to the condition or the number of consultants with expertise in the specific forms of ES, were not associated with ES rates for patients with benign gallbladder diseases^14^. Surgeon-led quality improvement initiatives, such as the Cholecystectomy Quality Improvement Collaborative (Chole-QuIC), have the potential to increase the uptake of ES^22^. Lessons from these initiatives, which warrant wider consideration, include the importance of ensuring that all stakeholders (surgeons, senior service managers, and staff gatekeeping emergency theatre lists) agree on the purpose and benefits of rapid surgical intervention^22^.

For acute appendicitis and abdominal wall hernia, the level of unexplained variation in ES rates across trusts was moderately high and may reflect the lack of evidence on which patients benefit from ES *versus* NES for these conditions, that there are fewer well defined care pathways, and a lack of clinical guidelines in the UK to inform the choice of whether or not a patient has ES^23^^,^^24^. It is also notable that in this study, abdominal wall hernia covered heterogenous diagnostic subgroups that comprised inguinal, femoral, umbilical, and ventral hernias, as well as bilateral hernias. The unexplained variation across trusts remained after adjusting for these diagnostic subcategories. It is also important to recognize that over the study period, emergency admissions with abdominal wall hernia were not managed by a distinct surgical subspecialty in the UK, which may have hindered attempts to standardize practice^24^, and that different local policies on restricting elective hernia surgery affected emergency provision^8^^,^^25^. For patients with uncomplicated acute appendicitis, the emerging evidence for use of antibiotics as an alternative to ES may explain the variability^6^^,^^7^.

For patients with intestinal obstruction and diverticular disease, the variation in ES rates across trusts was relatively low, which, for diverticular disease, may reflect increased standardization in clinical management of the condition over the study period, that RCTs were undertaken, and that these required clinical pathways to be developed^26–28^. For diverticular disease, there is consensus in the UK on the surgical specialty that manages patients, that is colorectal surgery. For patients with acute diverticular disease, the rate of ES has declined over time^29^, and the low ES rate reflects current NICE recommendations that encourages NES strategies and the lack of high-quality evidence on the effectiveness of ES for patients with acute diverticular disease^30^. Indeed, for both these conditions, the unexplained variation in ES across trusts, while low compared to the other three conditions, is still of sufficient magnitude to raise concerns that for some underlying patient subgroups, patients with similar acute presentations would receive ES in some trusts and NES in others.

This research extends previous studies which have generally found lower rates of ES for specific subgroups of older patients presenting with acute conditions in the UK^12–14^. Reports by the Royal College of Surgeons of England have generally found lower levels of ES for patients aged over 75 *versus* those aged 65–74, notably for patients with acute cholelithiasis^13^^,^^14^. These previous national recommendations have discouraged ES rationing by biological age^13^^,^^14^ and called for further research on ES by age to also consider co-morbidities and frailty. A previous study of five common surgical emergencies, including appendicitis and incarcerated or strangulated hernia, found that rates of ES were lower in the UK than for comparable patients in the United States, and that in the UK in-hospital mortality was lower for patients who had ES *versus* those who did not, after adjustment for some case-measures^31^. The current study found that even after adjusting for a wider range of case mix measures, including frailty, the rate of ES generally decreased with increasing age. For patients with acute cholelithiasis and appendicitis, this age gradient was especially steep and went across the age distribution. For patients presenting with hernia, previous studies reported higher ES rates for patients aged over 75 *versus* patients aged 65–74^12^^,^^13^.

Previous reports comparing the rate of ES across age groups^12^^,^^13^ did not adjust for differences in levels of frailty, number of co-morbidities, or diagnostic subcategories. The current finding of a strong association between increasing frailty and ES receipt in patients with no Charlson co-morbidities presenting with diverticular disease and intestinal obstruction might appear counterintuitive. However, a previous study on perforated diverticular disease found that while co-morbidity was associated with higher mortality, the relative risk of mortality, compared to the general population, was highest for patients without co-morbidity^32^. The present study emphasized the importance of allowing for other case mix differences, when trying to understand the reasons for different rates of ES across patient groups. Quality improvement initiatives, such as the National Emergency Laparotomy Audit (NELA) and the Emergency Laparotomy Pathway–Quality Improvement Care Bundle (ELPQuick), have highlighted clinical practice variations, and encouraged improvements in the quality of ES provision^33^, but have not considered those patients who do not have ES.

This study has several strengths. First, the study considered all eligible emergency admissions from 136 acute hospital trusts across a 10-year period. By adopting these broad inclusion criteria, the study had a representative sample of emergency admissions that was sufficiently large to draw inferences about the association of a multitude of routinely measured patient factors with receipt of ES. Second, unlike previous comparisons of ES rates across areas and patient demographics^11–13^, the study was able to adjust for differences in other routinely available measures of patient case mix, in particular patient frailty and the number of co-morbidities. Third, the study used clinically relevant definitions of ES that could be applied to large-scale administrative data sets.

The limitations of this paper include the following. Detailed information on patients’ acute condition, for example information on their physiology, and for some conditions (for example, acute appendicitis, diverticular disease) the lack of information from imaging could mean that differences in the true diagnosis or severity of the condition may explain some of the variations in ES across NHS trusts or patient subgroups. Other unmeasured variables which could be important in helping understand these variations include patient preferences and lack of emergency theatre capacity for these conditions within the local healthcare systems. A further challenge is that the categorization of ES *versus* NES assumes accurate coding of OPCS procedures and episode dates. It is conceivable that there were coding differences across NHS trusts; for example, some trusts could code patients with umbilical hernia as ventral hernia, and vice versa. However, this would seem unlikely to explain differences in ES rates of the magnitude reported. Third, this paper does not contrast outcomes between patients who received ES *versus* those who had NES, or according to NHS hospital trusts with different rates of ES. The paper does not therefore attempt to define the optimum level of ES *versus* NES for each condition.

This paper therefore highlights important areas for further research. In particular, given the wide variations in ES rates reported, there is a clear requirement for evidence on the effectiveness of ES *versus* NES strategies for subgroups of patients presenting as emergency admissions with acute conditions. The ESORT study will use the variation in ES rates across NHS trusts and hospitals to assess the effectiveness and cost-effectiveness of ES for each of these five conditions^15^. This can provide complementary evidence to that available from recent RCTs^6^ and ongoing observational studies^24^^,^^34^.

## Funding

The ESORT study is funded by the National Institute for Health Research Health Services and Delivery Research (project number 18/02/25). This report is independent research supported by the National Institute for Health Research ARC North Thames. The views expressed in this publication are those of the author(s) and not necessarily those of the National Institute for Health Research or the Department of Health and Social Care.

## Supplementary Material

zrab094_Supplementary_Data
