# Supplementary material for: Variation in the rates of emergency surgery amongst emergency admissions to hospital for common acute conditions
Source: BJS Open. 2021 Nov 17;5(6):zrab094. doi: 10.1093/bjsopen/zrab094 (PMC8599905; doi:10.1093/bjsopen/zrab094)
Supplement: zrab094_Supplementary_Data [file zrab094_supplementary_data.docx]

**Supplementary information**

**Variation in the rates of emergency surgery amongst emergency admissions to hospital for common acute conditions**

**Clinical panel defined inclusion and exclusion criteria**

A clinical panel of 11 surgeons (four women) and one anaesthetist, based in 12 different UK centres across 11 different regions within the UK, that included clinicians with subspecialty interests was convened, and met twice.^39^ A list of ICD10 codes for potential inclusion criteria and potential exclusion diagnoses was presented following the first facilitated meeting and discussed at the second (see supplementary Table 1). Each panellist privately rated the potential criteria. A relevant diagnosis for inclusion required at least 75% panel support and diagnoses forming exclusion criteria required at least 25% panel support. The consensus from the clinical panel with respect to changes to the inclusion and exclusion criteria are listed in Supplementary Table 2.

**Clinical panel definition of Emergency Surgery**

The panel also defined the procedures that represented ‘emergency surgery’ for patients in each cohort using a Delphi process. A list of Office of Population, Censuses and Surveys classification of surgical operations and procedures (OPCS) codes for potentially relevant procedures for each condition was drawn up and discussed at the first meeting. Panellists indicated privately whether each procedure constituted emergency surgery, and stated the number of days (e.g. three days, five days, seven days etc) within which surgery must be undertaken to count as ‘emergency surgery’. Anonymised panel results were discussed at the second meeting before re-rating. A procedure was defined as constituting ‘emergency surgery’ if according to at least 50% of the panelists the specific procedure met that definition, and it was undertaken within the requisite number of days to meet the criteria for ‘emergency surgery’ according to the median stipulated across the members of the clinical panel (for agreed definition of ES, see results).

According to the consensus of the clinical panel, the defined time window for ES was within three days (hernia), seven days (appendicitis, cholelithiasis, intestinal obstruction), or any time within the index emergency admission (diverticular disease) (supplementary table 3).

**Supplementary Table 1: List of ICD-10 codes considered for inclusion criteria**

| **Population** | **ICD-10** | **Description** |
| --- | --- | --- |
| Appendicitis | K35 | Acute appendicitis |
|  | K35.2 | Acute appendicitis with generalized peritonitis |
|  | K35.3 | Acute appendicitis with localized peritonitis |
|  | K35.8 | Acute appendicitis, other and unspecified |
|  | K37 | Unspecified appendicitis |
|  |  |  |
| Diverticular disease | K57.0 | Diverticular disease of small intestine with perforation and abscess |
|  | K57.1 | Diverticular disease of small intestine without perforation or abscess |
|  | K57.2 | Diverticular disease of large intestine with perforation and abscess |
|  | K57.3 | Diverticular disease of large intestine without perforation or abscess |
|  | K57.4 | Diverticular disease of both small and large intestine with perforation and abscess |
|  | K57.5 | Diverticular disease of both small and large intestine without perforation or abscess |
|  | K57.8 | Diverticular disease of intestine, part unspecified, with perforation and abscess |
|  | K57.9 | Diverticular disease of intestine, part unspecified, without perforation or abscess |
|  |  |  |
| Cholelithiasis | K80.0 | Calculus of gallbladder with acute cholecystitis |
|  | K80.1 | Calculus of gallbladder with other cholecystitis |
|  | K80.2 | Calculus of gallbladder without cholecystitis |
|  | K80.3 | Calculus of bile duct with cholangitis |
|  | K80.4 | Calculus of bile duct with cholecystitis |
|  | K80.5 | Calculus of bile duct without cholangitis or cholecystitis |
|  | K80.8 | Other cholelithiasis |
|  |  |  |
| Hernia | K40.0 | Bilateral inguinal hernia, with obstruction, without gangrene |
|  | K40.1 | Bilateral inguinal hernia, with gangrene |
|  | K40.2 | Bilateral inguinal hernia, without obstruction or gangrene |
|  | K40.3 | Unilateral or unspecified inguinal hernia, with obstruction, without gangrene |
|  | K40.4 | Unilateral or unspecified inguinal hernia, with gangrene |
|  | K40.9 | Unilateral or unspecified inguinal hernia, without obstruction or gangrene |
|  | K41.0 | Bilateral femoral hernia, with obstruction, without gangrene |
|  | K41.1 | Bilateral femoral hernia, with gangrene |
|  | K41.2 | Bilateral femoral hernia, without obstruction or gangrene |
|  | K41.3 | Unilateral or unspecified femoral hernia, with obstruction, without gangrene |
|  | K41.4 | Unilateral or unspecified femoral hernia, with gangrene |
|  | K41.9 | Unilateral or unspecified femoral hernia, without obstruction or gangrene |
|  | K42.0 | Umbilical hernia with obstruction, without gangrene |
|  | K42.1 | Umbilical hernia with gangrene |
|  | K42.9 | Umbilical hernia without obstruction or gangrene |
|  | K43.0 | Incisional hernia with obstruction, without gangrene |
|  | K43.1 | Incisional hernia with gangrene |
|  | K43.2 | Incisional hernia without obstruction or gangrene |
|  | K43.3 | Parastomal hernia with obstruction, without gangrene |
|  | K43.4 | Parastomal hernia with gangrene |
|  | K43.5 | Parastomal hernia without obstruction or gangrene |
|  | K43.6 | Other and unspecified ventral hernia with obstruction, without gangrene |
|  | K43.7 | Other and unspecified ventral hernia with gangrene |
|  | K43.9 | Other and unspecified ventral hernia without obstruction or gangrene |
|  |  |  |
| Intestinal obstruction | K56.0 | Paralytic ileus |
|  | K56.1 | Intussusception |
|  | K56.2 | Volvulus |
|  | K56.3 | Gallstone ileus |
|  | K56.4 | Other impaction of intestine |
|  | K56.5 | Intestinal adhesions [bands] with obstruction |
|  | K56.6 | Other and unspecified intestinal obstruction |
|  | K56.7 | Ileus, unspecified |

**Supplementary Table 2: Clinical panel-derived diagnostic inclusion and exclusion criteria**

| **Inclusion criteria** | **Appendicitis** | **Cholelithiasis** | **Diverticular disease** | **Hernia** | **Intestinal obstruction*** |
| --- | --- | --- | --- | --- | --- |
| Agreed for inclusion | All included | Calculus of gall bladder | Large intestine | Inguinal; Femoral; Umbilical; Ventral | Intestinal adhesions, Intussusception; Volvulus; Gallstone ileus; Other obstruction |
| Dropped from inclusion | None | Calculus of bile duct; Other cholelithiasis | Small intestine; Small and large intestine; Unspecified | Incisional; Parastomal | Paralytic ileus; Other impaction; Ileus, unspecified |
| **Exclusion criteria** |  |  |  |  |  |
| Agreed for exclusion | Pregnancy; Appendiceal cancer |  |  | Pregnancy; Ischaemia; Cancer. | Colorectal cancer with metastases; Gynaecological cancer; Ischaemia |
| Dropped for exclusion |  |  |  |  |  |

* inclusion allowed for a relevant diagnosis in the second diagnosis field provided the main diagnosis was non-metastatic colorectal cancer.

**Supplementary Table 3: Clinical panel-derived definitions of emergency surgery for each condition**

|  | **Appendicitis** | **Cholelithiasis** | **Diverticular disease** | **Hernia** | **Intestinal obstruction** |
| --- | --- | --- | --- | --- | --- |
| Procedures defined as ‘emergency surgery’ | 21 of 33 | 11 of 48 | 45 of 57 | 52 of 59 | 111 of 140 |
| Common procedures excluded from definition of ‘emergency surgery’ | *Unspecified other excision of appendix | Endoscopic sphincterotomy | Image controlled percutaneous drainage | none | none |
| Threshold for a procedure in the index admission to be ‘emergency surgery’ | 7 days | 7 days | Any time | 3 days | 7 days |
| Threshold for a procedure in a readmission to be ‘emergency surgery’ | 7 days | 7 days | 14 days | 3 days | 7 days |
| Emergency surgery: n (% of cohort) | 249,165 (91.4) | 52,543  (21.5) | 18,085  (12.8) | 62,578  (57.5) | 41,656  (29.7) |

**Supplementary table 4:** **Inclusion and exclusion criteria for emergency admissions to 136 acute NHS Trusts in England April 2010-December 2019**

|  | **Appendicitis** | **Cholelithiasis** | **Diverticular disease** | **Hernia** | **Intestinal obstruction** |
| --- | --- | --- | --- | --- | --- |
| **Meet inclusion criteria: n** | **308,915** | **367,497** | **200,816** | **147,122** | **238,097** |
| **Exclusions: n (%)** |  |  |  |  |  |
| No episode with a consultant surgeon | 8,582 (2.8%) | 56,913 (15.5%) | 26,606 (13.2%) | 9,457 (6.4%) | 34,475 (14.5%) |
| No eligible diagnosis in the first two episodes | 1,492 (0.5%) | 5,593 (1.5%) | 5,501 (2.7%) | 1,761 (1.2%) | 7,208 (3.0%) |
| Not admitted through A&E or GP | 21,053 (6.8%) | 21,637 (5.9%) | 12,182 (6.1%) | 11,477 (7.8%) | 13,595 (5.7%) |
| Clinical panel exclusion criteria | 1,443 (0.5%) | n/a | n/a | 7,574 (5.1%) | 10,512 (4.4%) |
| Missing discharge data | 351 (0.1%) | 274 (0.1%) | 146 (0.1%) | 115 ((0.1%) | 340 (0.1%) |
| Ineligible hospital | 1,007 (0.3%) | 1,648 (0.4%) | 768 (0.4%) | 490 (0.3%) | 911 (0.4%) |
| Transfer between hospitals before index episode | 634 (0.2%) | 588 (0.2%) | 406 (0.2%) | 173 (0.1%) | 503 (0.2%) |
| Other admission meeting inclusion criteria in previous 12 months | 3,572 (1.2%) | 38,812 (10.6%) | 15,544 (7.7%) | 7,908 (5.4%) | 31,686 (13.3%) |
| Emergency surgery prior to index episode | 2,519 (0.8%) | 406 (0.1%) | 574 (0.3%) | 842 (0.6%) | 1,123 (0.5%) |
| **Included in cohort: n** | **268,253** | **241,626** | **139,090** | **107,325** | **137,744** |
| Emergency surgery: n (%) |  |  |  |  |  |
| Yes | 247,475 (92.3%) | 52,014 (21.5%) | 15,255 (11.0%) | 62,150 (57.9%) | 41,144 (29.9%) |
| No | 20,778 (7.7%) | 189,612 (78.5%) | 123,835 (89.0%) | 45,175 (42.1%) | 96,600 (70.1%) |

**Supplementary Table 5: Diagnostic subcategories**

| **Population** | **ICD-10** | **Description** | **N (%)** |
| --- | --- | --- | --- |
| Appendicitis | K35 | Acute appendicitis | 14,810 (5.5) |
|  | K35.2 | Acute appendicitis with generalized peritonitis | 10,482 (3.9) |
|  | K35.3 | Acute appendicitis with localized peritonitis | 59,465 (22.2) |
|  | K35.8 | Acute appendicitis, other and unspecified | 147,103 (54.8) |
|  | K37 | Unspecified appendicitis | 36,393 (13.6) |
|  |  |  |  |
| Diverticular disease | K57.2 | Diverticular disease of large intestine with perforation and abscess | 32,741 (23.8) |
|  | K57.3 | Diverticular disease of large intestine without perforation or abscess | 106,349 (77.2) |
|  |  |  |  |
| Cholelithiasis | K80.0 | Calculus of gallbladder with acute cholecystitis | 87,021 (36.0) |
|  | K80.1 | Calculus of gallbladder with other cholecystitis | 67,697 (28.0) |
|  | K80.2 | Calculus of gallbladder without cholecystitis | 86,908 (36.0) |
|  |  |  |  |
| Hernia | K40.0 | Bilateral inguinal hernia, with obstruction, without gangrene | 962 (0.1) |
|  | K40.1 | Bilateral inguinal hernia, with gangrene | 52 (0.0) |
|  | K40.2 | Bilateral inguinal hernia, without obstruction or gangrene | 2,024 (1.9) |
|  | K40.3 | Unilateral or unspecified inguinal hernia, with obstruction, without gangrene | 17,274 (16.1) |
|  | K40.4 | Unilateral or unspecified inguinal hernia, with gangrene | 738 (0.7) |
|  | K40.9 | Unilateral or unspecified inguinal hernia, without obstruction or gangrene | 30,522 (28.4) |
|  | K41.0 | Bilateral femoral hernia, with obstruction, without gangrene | 263 (0.2) |
|  | K41.1 | Bilateral femoral hernia, with gangrene | 37 (0.0) |
|  | K41.2 | Bilateral femoral hernia, without obstruction or gangrene | 56 (0.1) |
|  | K41.3 | Unilateral or unspecified femoral hernia, with obstruction, without gangrene | 8,517 (7.9) |
|  | K41.4 | Unilateral or unspecified femoral hernia, with gangrene | 1,150 (1.1) |
|  | K41.9 | Unilateral or unspecified femoral hernia, without obstruction or gangrene | 3,498 (3.3) |
|  | K42.0 | Umbilical hernia with obstruction, without gangrene | 18,387 (17.1) |
|  | K42.1 | Umbilical hernia with gangrene | 1,248 (1.2) |
|  | K42.9 | Umbilical hernia without obstruction or gangrene | 20,417 (19.0) |
|  | K43.6 | Other and unspecified ventral hernia with obstruction, without gangrene | 2,083 (1.9) |
|  | K43.7 | Other and unspecified ventral hernia with gangrene | 97 (0.1) |
|  |  |  |  |
| Intestinal obstruction | K56.1 | Intussusception | 1,481 (1.1) |
|  | K56.2 | Volvulus | 16,277 (11.8) |
|  | K56.3 | Gallstone ileus | 2,045 (1.5) |
|  | K56.5 | Intestinal adhesions [bands] with obstruction | 47,737 (34.7) |
|  | K56.6 | Other and unspecified intestinal obstruction | 70,204 (51.0) |

**Supplementary Table 6: Characteristics of emergency surgery**

|  | **Appendicitis**  **(n=247,475)** | **Cholelithiasis**  **(n=52,014)** | **Diverticular disease (n=15,255)** | **Hernia**  **(n=62,150)** | **Intestinal obstruction (n=41,144)** |
| --- | --- | --- | --- | --- | --- |
| Day of surgery: median (IQR) | 1 (0;1) | 2 (1;4) | 1 (0;2) | 0 (0;1) | 1 (0;3) |
| Five most common main procedures: procedure [OPCS code] and n (%) | Emergency excision of abnormal appendix NEC [H012]  162,584 (65.7)    Unspecified other excision of appendix [H029]  42,864 (17.3)  Emergency excision of abnormal appendix and drainage HFQ [H011]  25,580 (10.3)  Unspecified emergency excision of appendix [H019]  9,086 (3.7)  Emergency excision of normal appendix [H013]  3,472 (1.4) | Total cholecystectomy NEC [J183]  44,614 (85.8)  Percutaneous drainage of gall bladder [J241]  2,354 (4.5)  Partial cholecystectomy NEC [J185]  1,755 (3.4)  Drainage of gall bladder [J212]  1,365 (2.6)  Total cholecystectomy and exploration of common  bile duct [J182]  1,281 (2.5) | Rectosigmoidectomy and closure of rectal stump and exteriorisation of bowel [H335]  8,883 (58.2)  Irrigation of peritoneal cavity [T463]  1,288 (8.4)  Sigmoid colectomy and exteriorisation of bowel NEC [H105]  1,044 (6.8)  Anterior resection of rectum and exteriorisation of bowel [H336]  497 (3.3)  Loop colostomy [H151]  360 (2.4) | Primary repair of inguinal hernia using insert of prosthetic material [T202]  18,993 (30.6)  Repair of umbilical hernia using sutures [T243]  12,349 (19.9)  Repair of umbilical hernia using insert of  prosthetic material [T242] 8,386 (13.5)  Primary repair of femoral hernia using sutures [T223]  6,304 (10.1)  Primary repair of femoral hernia using insert of prosthetic material [T222]  4,644 (7.5) | Freeing of adhesions of peritoneum [T413]  12,740 (31.0)  Ileectomy and anastomosis of ileum to ileum [G693]  3,522 (8.6)  Endoscopic division of adhesions of peritoneum [T423]  2,978 (7.2)  Division of band of peritoneum [T412]  2,133 (5.2)  Freeing of extensive adhesions of peritoneum [T415]  1,816 (4.4) |

**Supplementary Table 7: Adjusted odds ratios for association between SCARF frailty index and receipt of ES for diverticular disease and intestinal obstruction at each level of the Charlson comorbidity index**

|  | **Appendicitis**  **(n=268,253)** | **Cholelithiasis**  **(n=241,626)** | **Diverticular disease**  **(n=139,090)** | **Hernia**  **(n=107,325)** | **Intestinal obstruction**  **(n=137,744)** |
| --- | --- | --- | --- | --- | --- |
| Charlson comorbidity index: none | | | | | |
| Frailty index:  Fit  Mild  Moderate  Severe | - | - | reference  2.24 (2.10, 2.38)  5.07 (4.55, 5.64)  7.46 (6.34, 8.38) | - | reference  1.62 (1.56, 1.69)  2.49 (2.33, 2.66)  2.32 (2.10, 2.56) |
| Charlson comorbidity index: 1 | | | | | |
| Frailty index:  Fit  Mild  Moderate  Severe | - | - | reference  1.57 (1.42, 1.73)  2.55 (2.26, 2.87)  3.14 (2.67, 3.70) | - | reference  1.18 (1.11, 1.24)  1.57 (1.47, 1.68)  1.87 (1.71, 2.06) |
| Charlson comorbidity index: 2 | | | | | |
| Frailty index:  Fit  Mild  Moderate  Severe | - | - | reference  0.93 (0.71, 1.21)  1.13 (0.86, 1.47)  1.38 (1.03, 1.86) | - | reference  0.89 (0.79, 1.00)  1.04 (0.92, 1.17)  1.23 (1.07, 1.42) |
| Charlson comorbidity index: 3 or more | | | | | |
| Frailty index:  Fit  Mild  Moderate  Severe | - | - | reference  0.72 (0.33, 1.54)  0.86 (0.41, 1.80)  0.71 (0.33, 1.51) | - | reference  0.73 (0.51, 1.04)  0.67 (0.48, 0.95)  0.72 (0.50, 1.03) |
